# Additively Manufactured Patient-Specific Anthropomorphic Thorax Phantom With Realistic Radiation Attenuation Properties

**DOI:** 10.3389/fbioe.2020.00385

**Published:** 2020-05-08

**Authors:** Sepideh Hatamikia, Gunpreet Oberoi, Ewald Unger, Gernot Kronreif, Joachim Kettenbach, Martin Buschmann, Michael Figl, Barbara Knäusl, Francesco Moscato, Wolfgang Birkfellner

**Affiliations:** ^1^Austrian Center for Medical Innovation and Technology, Wiener Neustadt, Austria; ^2^Center for Medical Physics and Biomedical Engineering, Medical University of Vienna, Vienna, Austria; ^3^Institute of Diagnostic, Interventional Radiology and Nuclear Medicine, Landesklinikum Wiener Neustadt, Wiener Neustadt, Austria; ^4^Department of Radiation Oncology, Medical University of Vienna, Vienna, Austria; ^5^Ludwig Boltzmann Institute for Cardiovascular Research, Vienna, Austria

**Keywords:** additive manufacturing, 3D printed thorax, patient-specific phantom, computed tomography (CT) imaging, radiation attenuation, precision medicine

## Abstract

Conventional medical imaging phantoms are limited by simplified geometry and radiographic skeletal homogeneity, which confines their usability for image quality assessment and radiation dosimetry. These challenges can be addressed by additive manufacturing technology, colloquially called 3D printing, which provides accurate anatomical replication and flexibility in material manipulation. In this study, we used Computed Tomography (CT)-based modified PolyJet^TM^ 3D printing technology to print a hollow thorax phantom simulating skeletal morphology of the patient. To achieve realistic heterogenous skeletal radiation attenuation, we developed a novel radiopaque amalgamate constituting of epoxy, polypropylene and bone meal powder in twelve different ratios. We performed CT analysis for quantification of material radiodensity (in Hounsfield Units, HU) and for identification of specific compositions corresponding to the various skeletal structures in the thorax. We filled the skeletal structures with their respective radiopaque amalgamates. The phantom and isolated 3D printed rib specimens were rescanned by CT for reproducibility tests regarding verification of radiodensity and geometry. Our results showed that structural densities in the range of 42–705HU could be achieved. The radiodensity of the reconstructed phantom was comparable to the three skeletal structures investigated in a real patient thorax CT: ribs, ventral vertebral body and dorsal vertebral body. Reproducibility tests based on physical dimensional comparison between the patient and phantom CT-based segmentation displayed 97% of overlap in the range of 0.00–4.57 mm embracing the anatomical accuracy. Thus, the additively manufactured anthropomorphic thorax phantom opens new vistas for imaging- and radiation-based patient care in precision medicine.

## Introduction

Medical imaging phantoms are widely used in radiology and medical physics to evaluate and adjust the performance of imaging devices (Filippou and Tsoumpas, 2018). Their purpose is to assess imaging quality and radiation dosimetry by facilitating unrestricted repeated scans with defined parameters (Pogue and Patterson, 2006). Traditional mold phantoms usually consist of materials with tissue-equivalent radiopacity but they often have simple, homogenous forms and dimensions and do not mimic anatomy accurately (Ionita et al., 2014; Homolka et al., 2017). This often renders conclusions from phantoms to humans implausible (Pogue and Patterson, 2006; Jahnke et al., 2017; Hazelaar et al., 2018). To precisely analyze different imaging systems, a realistic phantom should accurately simulate a true patient scan including anatomy, tissue density and X-ray attenuation characteristics in the case of X-ray or computed tomography (CT) imaging. Tissue equivalency is not only relevant for image quality assessment but also for accurate radiation dosimetry especially for charged particles where the tissue composition influences the particle interactions (Kostiukhina et al., 2017; Kostiukhina et al., 2019). Despite the ubiquitous existence of various thoracic pathologies and their imaging-based surgical and diagnostic procedures, more realistic models are required (Nardi et al., 2017; Huang et al., 2019; Montigaud et al., 2019).

Various anthropomorphic models were implemented for the assessment of CT image quality in terms of contrast, spatial resolution, density and image-to-background noise (Wildgruber et al., 2016; Nardi et al., 2017; Witowski et al., 2019). By applying dedicated algorithms and protocols, the stability of the imaging system can be measured in dependence of the size of the object, position of different anatomic areas, dose levels and source-detector trajectories (Hatamikia et al., 2019a,b,c; Hernandez-Giron et al., 2019; Russ et al., 2020). Commercially available Alderson Rando anthropomorphic thorax phantoms (Phantom Laboratory, Salem, NY, United States) for X-ray and other diagnostic modalities consist of sections of human skeletons surrounded by tissue equivalent material (Wildgruber et al., 2016; Nardi et al., 2017). However, these phantoms are relatively expensive and not patient specific (Homolka et al., 2017; Filippou and Tsoumpas, 2018). For acquiring surgical skills human cadavers are conventionally used (Brouwers et al., 2018). Owing to the increasing ethical concerns regarding reduction, refinement and replacement of cadaver models it is imperative to develop patient-specific real size chest models of the thorax (Li et al., 2018; Király, 2019). Additive manufacturing is the process of assembling materials to create objects from a 3D model data by depositing materials normally layer upon layer. The availability of cost-effective 3-dimensional (3D) desktop printers offers new prospects for tailored phantoms to perform explicit clinical and research procedures (Hernandez-Giron et al., 2019; Ehler et al., 2014). CT-derived 3D printed anatomical models offer a new platform for quality control and precision medicine. In the past, fused deposition modeling (FDM) has been used to create custom-designed phantoms for patient specific dosimetric verification (Ehler et al., 2014). PolyJet^TM^ technology was later used to print a CT-based thorax phantom for radiation therapy evaluation (Mayer et al., 2015). Despite technological advances, these methods remain complex and rely on the principle of combining a limited number of materials and unrealistic Hounsfield units (HUs). HUs represent the linear attenuation coefficient of X-rays in CT; they are given in 12bit resolution and they are calibrated relative to water at room temperature. In this study, we propose a novel protocol to create an anthropomorphic thorax phantom with realistic (human-like) radiation attenuation properties in HUs based on CT data using PolyJet^TM^ printing technology. The proposed phantom can provide an illustrative anatomy to simulate dose exposure for clinical imaging in X-ray images as well as CT. Our protocol can be easily adopted to phantoms from other body parts such as pelvis, neck, hands and knees with specific CT-related image properties. Stemming from its unique fabrication process and custom design, this modified additive manufacturing process can be applied to various dosimetry approaches to measure the organ at risk dose for the respective procedure. We produced a modifiable modular phantom with the possibility to have different defects such as tumors at customized positions. This makes this phantom a suitable tool for 3D tomography reconstruction purposes in diagnosis and image-guided therapies. One of the recent interesting topics in this field is to perform target-based reconstruction for CT and Cone Beam CT (CBCT) in order to optimize source-detector trajectories (Hatamikia et al., 2019a, b, c; Stayman et al., 2019). The aim of this additively manufactured phantom and its design workflow is to give a reliable image quality at a specific region of interest. While it is unrealistic to have a dedicated phantom for each patient, being able to mimic various, also exotic anatomical conditions, is still a huge advantage for further development of imaging protocols and verification of advanced dose planning methods in radiation therapy.

With the opportunity to develop a phantom with target-specific lifelike anatomy we can verify these types of focused reconstructed images. As medical exposure to ionizing radiation is a well-known risk factor, equalizing the associated imaging dose and positioning accuracy is obligatory in radiographic diagnosis, image-guided-radiotherapy and interventions. This work aims to evaluate and benchmark the imaging dose and positioning accuracy needed for imaging thorax pathologies with the help of additively manufactured thorax model with true-to-life imaging properties. This phantom can also offer an advanced solution for education and skill development of medical professionals. Complex procedures and interventions can be trained based on such patient-specific 3D-printed phantoms and surgical procedures can be optimized in order to increase patient safety.

## Materials and Methods

### Three-Dimensional Phantom Design

**FIGURE 1:**
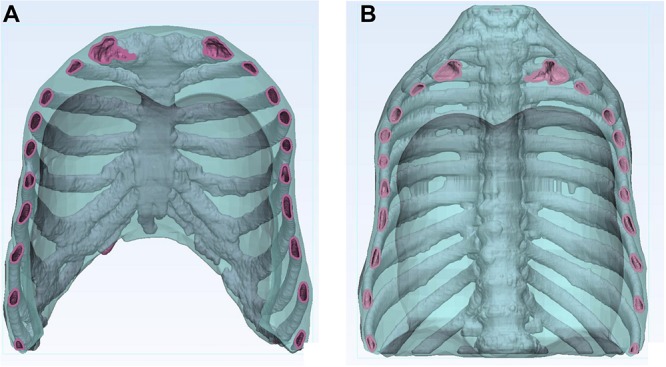
Two-dimensional image of the hollow thorax phantom. The images display the hollow skeletal integument (pink) consisting of ribs, sternum and vertebrae, embedded in a transparent body (blue). **(A)** Ventral segment, **(B)** Dorsal segment.

The study was approved by the Ethics Committee of the Medical University of Vienna (EK1253/2012). Anonymized patient CT data (SOMATOM Definition AS, Siemens Healthineers, Erlangen Germany) with the following parameters were employed in the study: tube voltage 120 kVp, tube current time product 315 mAs, slice thickness 2 mm including a total of 176 Digital Imaging and Communication in Medicine (DICOM) data files, representing axial slices through the body. The standard process of additively manufactured (AM) medical models was based on the direct segmentation of the bony parts from CT-based DICOM data set using Mimics software (Mimics USL 21.0, Materialize, Leuven, Belgium). A digital STL (Standard Tessellation Language) model was reconstructed from the DICOM data using 3-matic software (3-matic 13.0, Materialize, Leuven, Belgium). Using the same software, a 4mm thick skeletal integument was created by wrapping and hollowing functions specifically for sternum, ribs and vertebral column. The original patient dimensions and anatomy were preserved. The skeletal integument was then embedded digitally into a transparent body representing the intercostal and thoracic muscles, together called thorax phantom in this study. The skeletal integument was developed to serve as a framework for filling the inhomogeneous materials to create realistic radiation attenuation properties. Considering the limitation in the maximum printing dimensions, the STL file was divided into two parts coronally and processed for printing in two segments (ventral and dorsal) not to jeopardize the original patient thorax size (Figure 1).

### 3D-Printing Material Selection and Additive Manufacturing of the Thorax Phantom

The printable file in STL format was transported to a multi material PolyJet^TM^ printer Connex3 Objet500 (Stratasys, Eden Prairie, MN, United States of America) and a 1:1 scale model of skeletal integument was additively manufactured. The available 3D printed materials: rigid Vero pure white (RGD837) for the sternum, ribs and vertebral column; flexible Agilus30 Clear (FLX935) for embedding the skeletal integument and SUP706B as the support material were used to build the phantom. The printer settings were multi material mode with a standard layer thickness of 30 micrometer. The printing process of both print jobs took 120 h and needed 9,6 kg of flexible, 3,8 kg hard and 9,6 kg of support material, respectively. A rigorous cleaning procedure followed post printing, starting with manual removal of the support material from the hollow skeletal integument, alternating with waterjet (KK 30-VA, Krumm-Tec, Germany). To get the best results the model was then placed into a 2% NaOH solution, which gradually dissolved the support material, followed by a wash up with water. The above mentioned cleaning steps were repeated over a week to assure total removal of the support material from the alveolar skeletal integument making room for inserting heterogenous materials.

### Selection of Materials With Specific Radiation Attenuation Properties

**FIGURE 2:**
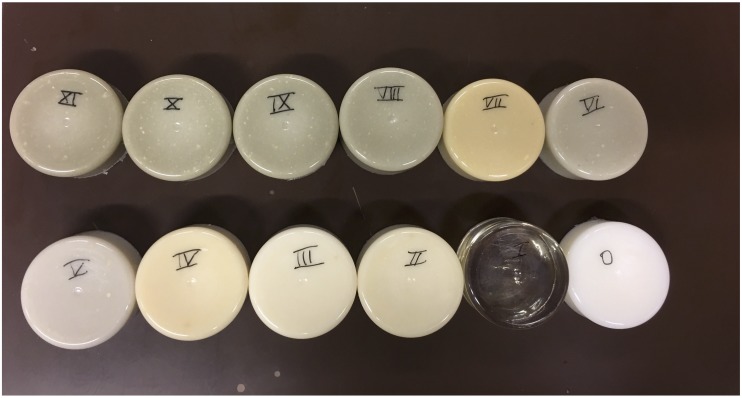
Radiopaque amalgamate samples used for analyzing radiodensities. The image displays cured radiopaque amalgamate samples in 12 different compositions labeled from 0 to XI.

A mixture of a three different materials (bone meal powder, epoxy and polypropylene powder) was used as radiopaque amalgamate to fill the skeletal integument representing sternum, ribs and vertebral column. The three components were mixed and analyzed in different compositions to mimic realistic HUs of different skeletal components of the thorax. However, twelve different compositions were created and tested to provide a wider range of density values for CT imaging for better reproducibility of phantoms from different body parts with different X-ray realistic attenuation values. We kept the samples at room temperature for more than a day until they were completely cured. The twelve different compositions of samples were labeled from 0, I, II, …, XI (Figure 2). To prepare the radiopaque amalgamate, we used a volume including 50% epoxy (containing 3:1 ratio of cast resin and hardener) and a 50% mixture of bone meal powder and polypropylene powder in different ratios.

The summation of bone meal and polypropylene powder is measured in volume in milliliter (ml) and is kept as equivalent to the epoxy volume in ml. Different proposed ratios of bone meal, epoxy and polypropylene powder related to the twelve radiopaque amalgamate samples labeled from 0, I, II, …, XI are presented in Table 1. The radiopaque samples were scanned using the same CT device and parameters described in Section “Three-dimensional phantom design.” We used the Analyze 12.0 toolkit (AnalyzeDirect, Overland Park, United States) to measure the HU related to each sample by taking line profiles at the defined regions of interest. The line profile gave the average and standard deviation over all points related to the selection.

**TABLE 1:** Different radiopaque amalgamate samples used in this study with their mixture ratios.

Sample	Bone meal/polypropylene ratio	Bone meal (%)	Epoxy (%)	Polypropylene (%)
0	–	–	50	50
I	–	–	100	–
II	1:5	8	50	42
III	1:4	10	50	40
IV	1:3	13	50	37
V	1:2	17	50	33
VI	1:1	25	50	25
VII	2:1	33	50	17
VIII	3:1	37	50	13
IX	4:1	40	50	10
X	5:1	42	50	8
XI	–	57	43	–

### Additively Manufactured Thorax Phantom and Filling Process

Following the aforementioned cleaning process, we injected the radiopaque amalgamation into the skeletal integument. We used mixtures IV, VII, and XI for the ventral vertebral body, rib cage including the sternum and dorsal vertebral body, respectively. Inlets were created on the dorsal surface of the sternum and at the caudal end of the dorsal vertebral body in the skeletal integuments using a rotary grinder (Dremel 4000, R. Bosch, United States) for injecting the proposed radiopaque amalgamate with a 50 ml syringe. First, we injected the mixture XI with the phantom in supine position until it entirely filled the dorsal vertebral body. This was confirmed by a CT scan and later set aside overnight for curing. The ventral vertebral body is composed of cancellous bone. In this study we replicated this bone structure by placing the liquid mixture IV in the vacuum device for almost 1 h. The long vacuuming led to the inclusion of very small air bubbles in the mixture which results in a cancellous structure. For the filling of the ventral vertebral body, we positioned the phantom cranio-caudally and injected the vacuumed mixture IV through the caudal inlet to avoid infiltration into the ribcage. After the inserted liquid was completely cured, we injected the mixture VII into the ribs and sternum through dorsal sternal inlet with the phantom in pronate position. Finally, the dorsal and ventral segments of the phantom were glued together with epoxy.

### Hounsfield Units for the Thorax Phantom

The thorax phantom underwent CT to evaluate the resulting HUs. We compared the resulting HUs of the patient CT and the thorax phantom CT in the ribs, ventral vertebral body and dorsal vertebral body. We computed the average and standard deviation using Analyze 12.0 (as explained in section “Selection of Materials With Specific Radiation Attenuation Properties”) from the patient and phantom CT and compared them for these three regions. Additionally, in order to establish the resemblance between our 3D printed thorax phantom with a commercially available thorax phantom we compared the radiation attenuation properties of our phantom with an Alderson Rando thorax phantom using CT with the same parameters as described in section “Three-dimensional phantom design.”

### Physical Dimensional Comparison Between the Phantom and Patient STL

**FIGURE 3:**
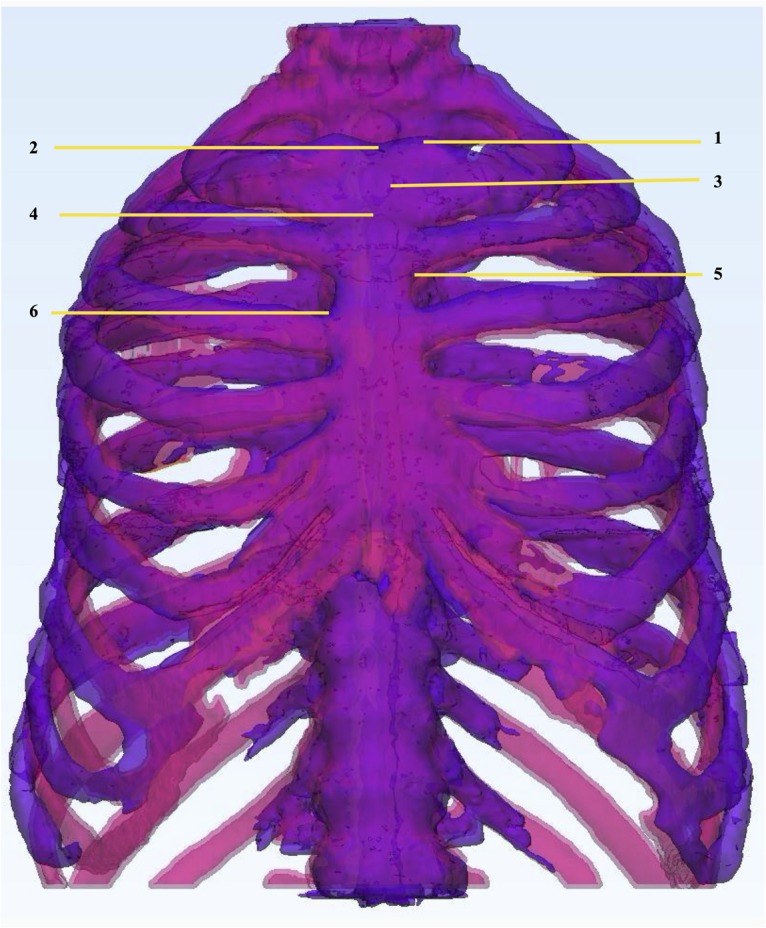
Physical dimensional comparison of 3D printed thorax phantom. Registration of thorax phantom STL (purple) on patient STL (pink) in 3-Matic 13.0 software using the following anatomic landmarks: (1) Clavicular notch, (2) Jugular notch, (3) Manubrium, (4) Sternal angle, (5) Superior articular angle, (6) Inferior articular angle.

To perform physical dimensional comparison between the thorax phantom and patient STL files we rescanned the additively manufactured thorax phantom using the same CT device (Section “Three-dimensional phantom design”) with tube voltage 120 kVp, tube current time product 270 mAs, slice thickness 1 mm and a total of 540 2D axial slices. We applied the same workflow for segmentation of the CT-based phantom imaging data as described in section “Three-dimensional phantom design” and created an STL file for dimensional comparison. We registered the phantom STL (purple) on the patient STL (pink) both manually and automatically in 3-matic software (Figure 3). We specified the following anatomical landmarks on both the STLs for manual registration: jugular notch, clavicular notch, manubrium and sternal angle on the sternum; most superior tip of the spinous process of the corresponding vertebral bodies; superior- and inferior- sternal and vertebral articular ends of the corresponding ribs. After manual anatomical registration we performed automatic global registration and processed the STLs for point-based part comparison analysis (PPCA), using an analysis tool available in 3-matic software for a detailed evaluation of the non-registered entities in PPCA, we applied segmentation function to the results from PPCA. This function classifies the non-registered entities into four categories, labeled as green (I), yellow (II), orange (III), and red (IV), for maximum to minimum registration.

### Reproducibility Tests for the Proposed Mixture and the Model Geometry

**FIGURE 4:**
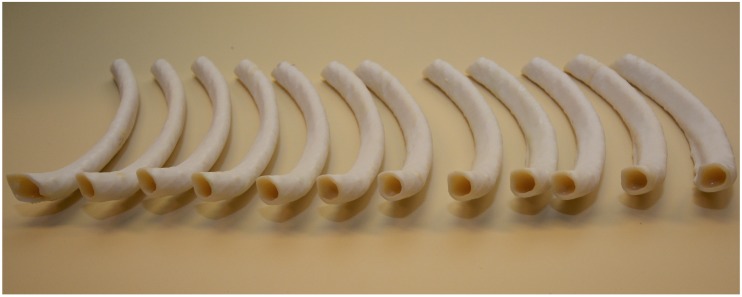
Twelve 3D Printed rib specimens. Twelve 3D Printed replicates of the dorsal segment from the body of rib 7.

For a detailed investigation of the reproducibility of the proposed mixture and the model geometry, we printed twelve replicas (Figure 4) of the dorsal segment from the body of rib seven following the same protocol as described in section “Three-dimensional phantom design.” The ribs were thoroughly cleaned (section “3D-printing material selection and additive manufacturing of the thorax phantom”). Radiopaque mixture VII was prepared individually for each replicate and after filling and curing, the replicated ribs underwent a CT scaning using the same parameters (section “Three-dimensional phantom design”). For comparative analysis of radiologic attenuation of the mixture and physical dimensional comparison we applied the same protocol as developed for thorax phantom (section “Hounsfield Units for the thorax phantom” and “Physical dimensional comparison between the phantom and patient STL”).

## Results

### Additively Manufactured Thorax Phantom

**FIGURE 5:**
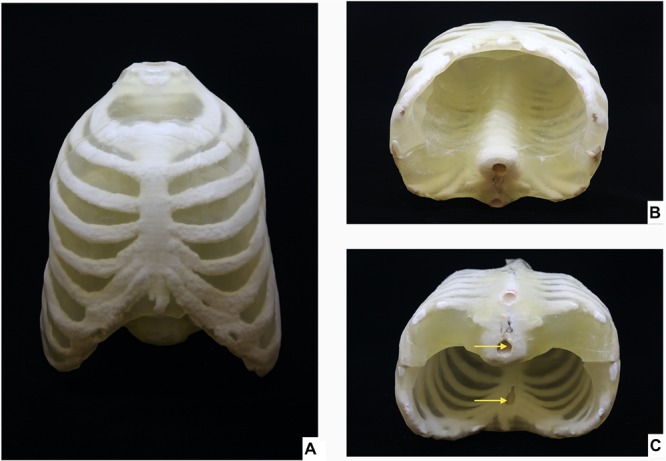
3D Printed thorax phantom. Thorax phantom consisting of the skeletal integument embedded in the transparent 3D printed body **(A)** Ventral view, **(B)** Caudal view of thorax phantom in supine position, **(C)** Caudal view of thorax phantom is prone position showing inlets (indicated by yellow arrows) for filling the radiopaque amalgamate in the sternum and ventral vertebral body.

Figures 5A–C show the 3D printed skeletal integument embedded in a transparent body. Inlets for filling the hollow skeletal component are shown in the Figure 5C.

### Resulting Radiopaque Amalgamate Samples With Specific Radiation Attenuation Properties

**FIGURE 6:**
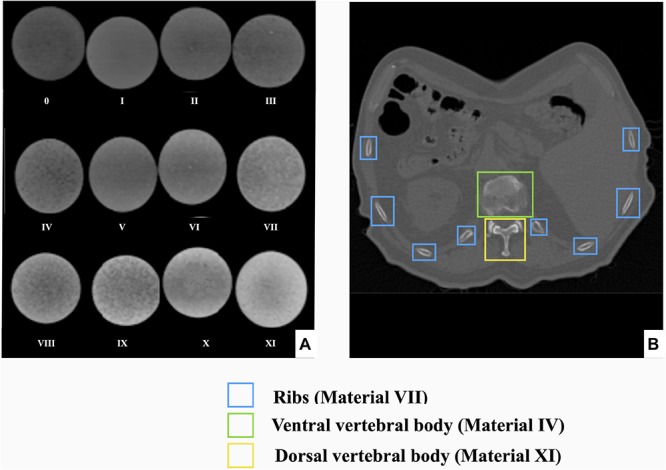
Radiodensity analysis of radiopaque amalgamate samples and their corresponding anatomic structures in human thorax. **(A)** CT scan of 12 different compositions of the cured radiopaque amalgamates for radiodensity analysis, **(B)** Axial section of CT from a patient thorax displaying that the radiodensities of the ribs, ventral and dorsal vertebral bodies were replicated by radiopaque amalgamates VII, IV, and XI, respectively.

We used the Analyze 12.0 toolkit to measure the radiodensity of the twelve different radiopaque amalgamates in the corresponding CT (Figure 6A). The resulting HUs ranging from 42 to 705 is presented in Table 2. Radiodensities of the ribs, ventral and dorsal vertebral bodies in the patient thorax CT were replicated by radiopaque amalgamates VII, IV, and XI, respectively, in the 3D printed phantom (Figure 6B).

**TABLE 2:** Resulting HUs related to twelve different radiopaque amalgamate samples used in this study.

Sample	Hounsfield unit (HU) (average ± standard deviation)
0	42 ± 20
I	134 ± 13
II	80 ± 56
III	135 ± 35
IV	215 ± 38
V	280 ± 40
VI	310 ± 53
VII	430 ± 35
VIII	485 ± 64
IX	530 ± 50
X	558 ± 26
XI	705 ± 66

### Resulting Density Values for the Thorax Phantom

**FIGURE 7:**
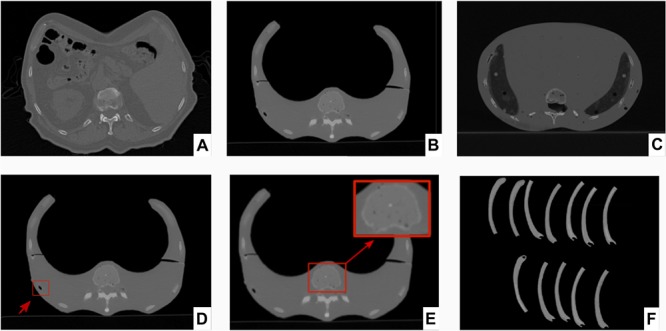
Corresponding axial sections from CT scans of patient, 3D printed thorax phantom and Alderson phantom and twelve printed ribs. Axial sections from CT scans of **(A)** Patient, **(B)** 3D printed thorax phantom, **(C)** Alderson Rando anthropomorphic thorax phantom. Axial sections from CT scans of 3D printed thorax phantom showing **(D)** Incorporation of air bubble (indicated by red arrow and box), **(E)** Replication of inhomogeneity in the ventral vertebral cancellous bone (magnified view in the red box), **(F)** CT scan of the rib specimens for reproducibility tests of the radiopaque amalgamate and model geometry.

**FIGURE 8:**
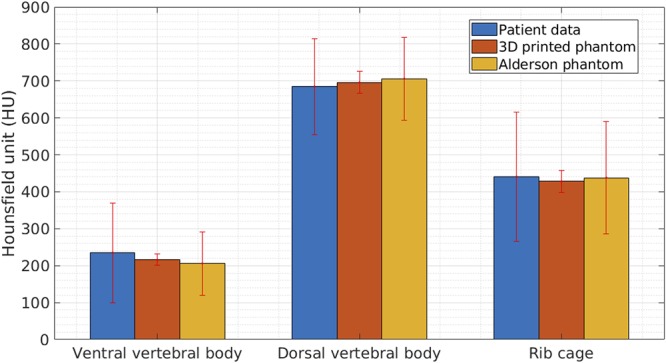
Results from comparison of radiodensities of patient, 3D printed thorax and Alderson Rando phantom. Graph showing average and standard deviation in radiodensities (represented by Hounsfield Units, HU) of the three anatomic structures, analyzed by CT scan of patient, 3D printed thorax and Alderson Rando phantom.

Corresponding axial sections from CT scans of patient, 3D printed thorax phantom and Alderson phantom are represented for comparison of radiodensity equivalence (Figures 7A–C). Based on the radiodensity analysis, the average density values achieved by the Analyze 12.0 toolkit for the phantom CT at ventral vertebral body, dorsal vertebral body and the rib cage were 216, 696, and 428 HU, respectively. For the real patient CT, for the same three areas the average density values achieved were 235, 685, and 441 HU, respectively. For Alderson phantom the corresponding average density values achieved were 206, 706, and 438 HU, respectively. The average and standard deviation of HUs achieved from patient, phantom CT and Alderson phantom at these three different bony areas are shown in Figure 8.

### Evaluation of the Geometric Accuracy of the Model

**FIGURE 9:**
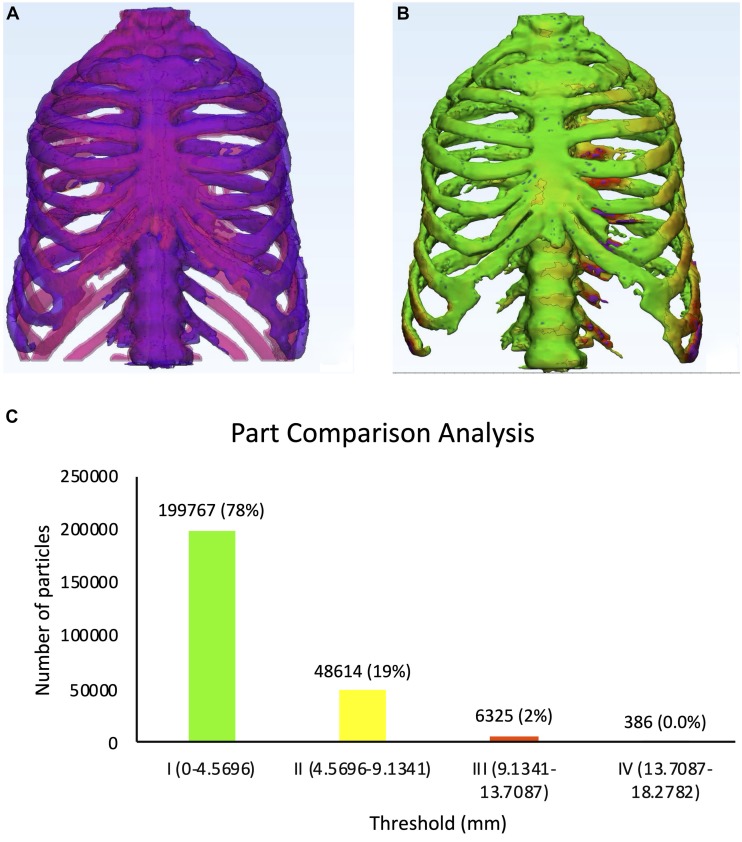
Physical dimensional comparison of 3D printed thorax phantom with the patient **(A)** Registration of a CT-derived STL of the 3D printed thorax phantom (purple) on the patient thorax CT-derived STL(pink) using anatomic landmarks in 3-Matic 13.0 software, **(B)** Color-coded result of Part comparison analysis, **(C)** Graph explaining the color-coding based quantification of element overlap; 78% elements on the patient thorax overlap with the 3D printed thorax phantom.

We compared the physical dimension of 3D printed thorax phantom with the patient after registration of the CT-derived STL of the 3D printed thorax phantom on the patient thorax CT-derived STL using anatomic landmarks in 3-Matic 13.0 software (Figure 9A).

Results were displayed as color-coded element overlap during PPCA and a graph explaining the color-coding based quantification of element overlap (Figures 9B,C). Number and percentage of the overlapped particles related to the PPCA analysis between the STL files acquired from patient and phantom thorax CT are given in Table 3. The results from PPCA show that an average of 78% and 19% of all entities belonging to the thresholds I and II, respectively, completely matched between the STL files acquired from patient and phantom thorax CT.

**TABLE 3:** Detailed description of the histogram of segmentation analysis of the PPCA between the STL files acquired from patient and phantom thorax CT.

Threshold (mm)	I (0–4.5696)	II (4.5696–9.1341)	III (9.1341–13.7087)	IV (13.7087–18.2782)
No. of overlapping particles	199767	48614	6325	386
% of overlapping particles	78	19	2	0

### Reproducibility Tests Results for the Bone Equivalent Amalgamate

We acquired a CT scan (section “Three-dimensional phantom design”) from the twelve rib samples (Figure 7F) which were prepared (section 2.7). In order to evaluate the resulting density values we used Analyze 12.0 (section 2.3). For each rib, we calculated the average for three different areas along the rib. Therefore, for all twelve rib samples we evaluated 36 values for the densities. Their mean HU value was found to be 436 ± 12HU reproducing the original average of 441HU achieved for the real patient (section “Resulting density values for the thorax phantom”).

**FIGURE 10:**
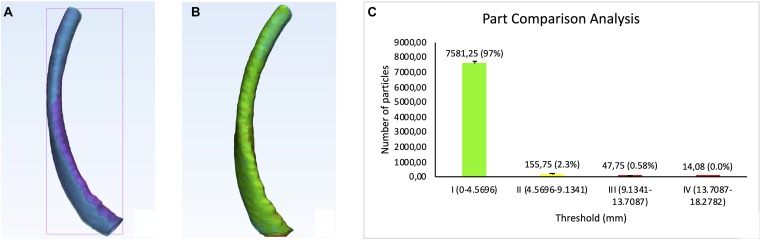
Physical dimensional comparison of 3D printed rib specimens. **(A)** Registration of one of the twelve CT-derived STLs of the 3D printed rib specimens (purple) on the patient thorax CT-derived STL (pink) using anatomic landmarks in 3-Matic 13.0 software, **(B)** Color-coded result of Part comparison analysis, **(C)** Graph explaining the color-coding based quantification of element overlap; 97% elements on the patient rib overlap with the 3D printed ribs.

We compared the physical dimension of 3D printed rib specimens after registration of one of the twelve CT-derived STLs of the 3D printed rib specimens on the patient thorax CT-derived STL using anatomic landmarks in 3-Matic 13.0 software (Figure 10A). Results were displayed as color-coded element overlap during PPCA and a graph explaining the color-coding based quantification of element overlap (Figures 10B,C). Number and percentage of the overlapping particles related to PPCA analysis between the STL files acquired from patient and the twelve phantom ribs are given in Table 4. We calculated the average over the particle percentages related to all twelve ribs and 97.08, 2.33, 0.58, 0% were achieved for threshold I, II, III, and IV, respectively. These results showed that an average of 97% of all entities belonging to the threshold I were completely matched for all twelve ribs. This confirms an accurate dimensional reproduction in the proposed 3D printing phantom.

**TABLE 4:** Detailed description of the histogram of segmentation analysis of the PPCA between the STL files acquired from the patient and twelve phantom ribs CT.

Threshold		I (0–4.5696)	II (4.5696–9.1341)	III (9.1341–13.7087)	IV (13.7087–18.2782)
Rib 1	Overlapped particle number # percentage (%)	7638 (97)	149 (2)	35 (0)	18 (0)
Rib 2	Overlapped particle number # percentage (%)	7659 (98)	137 (2)	27 (0)	11 (0)
Rib 3	Overlapped particle number # percentage (%)	7469 (97)	199 (2)	49 (1)	14 (0)
Rib 4	Overlapped particle number # percentage (%)	7270 (95)	317 (5)	26 (0)	13 (0)
Rib 5	Overlapped particle number # percentage (%)	7412 (95)	290 (4)	68 (1)	18 (0)
Rib 6	Overlapped particle number # percentage (%)	7818 (98)	66 (1)	43 (1)	17 (0)
Rib 7	Overlapped particle number # percentage (%)	7505 (98)	113 (2)	38 (0)	8 (0)
Rib 8	Overlapped particle number # percentage (%)	7742 (98)	84 (1)	48 (1)	20 (0)
Rib 9	Overlapped particle number # percentage (%)	7594 (98)	78 (2)	36 (0)	10 (0)
Rib 10	Overlapped particle number # percentage (%)	7683 (98)	83 (2)	39 (0)	11 (0)
Rib 11	Overlapped particle number # percentage (%)	7466 (96)	206 (3)	81 (2)	27 (0)
Rib 12	Overlapped particle number # percentage (%)	7719 (97)	147 (2)	83 (1)	2 (0)
Mean		7581.23 (97.08)	155.75 (2.33)	30.36 (0.58)	14.08 (0)

### Mimicking the Spongy Structure for Ventral Vertebral Body

The long vacuuming time was performed to add very small bubbles to the entire IV mixture which results in a spongiosum structure after curing. This mimics the cancellous bone structure. Figure 7. E represents the spongy structure achieved for the ventral vertebral.

## Limitation

The main limitation of this work is the operator dependent artifact production such as air bubbles during the filling process. One example of such an air bubble is shown in Figure 7D. D. We observed couple of air bubbles inside the entire phantom which are formed due to limited access to the inner areas inside the phantom. In order to overcome this limitation, we can place the whole phantom inside the vacuum device for a short time in order to remove air bubbles. The other solution can be to divide the phantom into smaller segments/volumes instead of two big parts which gives a better access to the inner part of the phantom resulting in a more accurate filling process.

## Discussion and Conclusion

CT and radiation therapy phantoms are required for test scans, device calibration, quality assurance, dosimetry, staff training and demonstration purposes. The production workflow of our novel thorax imaging model was different from the standard workflow of other human CT/magnetic resonance imaging (MRI) based AM medical models (Morikawa et al., 2017). The bottle-neck in such models is that they lack the possibility to control the material properties and consequently result in unrealistic homogenous HU. Our model solved this problem with a mold, created by a wrapped shell around the reconstructed bone and then injecting the region-specific radiopaque mixture with different HUs into the defined anatomic skeletal structures. We produced a modifiable modular phantom with the possibility to have different tumors and lesions at variable positions. The phantom in our study replicates the original thorax CT from the patient and can be applied in education of image-guided interventions in lung or for the source-detector trajectory optimization for target-based CBCT reconstruction (Hatamikia et al., 2019a,b,c; Stayman et al., 2019). The geometrical accuracy test results showed a good match between the digital model created by the patient CT and the model from the 3D printed phantom CT. This embraced the fact that the production of the phantom was not only simple and fast but accurate and reliable. Our results indicated that the defined workflow is suitable for any desired form or patient template. The first feasibility test showed good agreement between different density values of the proposed phantom to the initial patient CT scan as well as an Alderson thorax phantom CT scan within three different areas containing bony structure. We observe a high standard deviation of HU achieved for the patient CT specially at ventral vertebral body and rib cage compared to the phantom data (Figure 8). This high standard deviation arises from the fact that both the ventral vertebral body and rib cage have a cancellous (spongy) structure at the center and a very dense structure around which represents a very different radiation attenuation properties and therefore different HUs in those areas.

In this study, there was no possibility to create such a dual structure with our phantom design protocol. Therefore, we found the average HUs over both dense and spongy areas observed in the patient data for ventral vertebral body and rib cage and tried to mimic the average HU value for the corresponding parts in the phantom. Phantom consistency over time has not yet been demonstrated and is subject to future studies. Density value reproduction for the different bony structures is a future prospect of our work. Our study provides new possibilities for other research groups to develop their own customized phantom with the required radiation attenuation properties using a fast and cost-effective protocol. In addition to the air bubble problem (as illustrated in section “Limitation”) there are some other limitations in our presented method. First, the 3D printer specifications restrict the size of the printable phantom. For larger sized body regions, the phantom must be divided into several sections before being printed and glued together. This is the reason that we observed some misregistrations between the STL files acquired from patient and phantom thorax CT specially at caudal parts of the model (Figures 9A,B). The dorsal and ventral segments of the phantom model were placed into 2% NaOH in order to wash out the whole support material. This caused some deformation in the transparent body tissue which eventually led to misalliance while gluing the two segments with epoxy together. Second, the 3D printed integument affects image quality and phantom stability. In very thin sections, the 3D printing material of the shell is fragile, so it is necessary to accurately estimate the shell thickness according to the manufacturing method and experimental requirements.

Our results showed that a PolyJet^TM^ printer can be successfully used in combination with the modified segmentation workflow and additional materials to produce three-dimensional objects with the shape and radiation attenuation characteristics of patient bony structures. Although, the production of such phantom is simple and convenient, further exploration and improvement is needed for large-scale clinical applications.

## Data Availability Statement

The datasets generated for this study will not be made publicly available as the data consists of patient data.

## Ethics Statement

Written informed consent was obtained from the individual(s) for the publication of any potentially identifiable images or data included in this article.

## Author Contributions

SH, GO, and WB were involved in the study design, literature research, the data analysis, writing the manuscript. GK, JK, MB, MF, BK, and FM were involved in the study design and manuscript preparation. SH and EU was involved in study design, the data analysis, writing, and submission of the manuscript.

## Conflict of Interest

The authors declare that the research was conducted in the absence of any commercial or financial relationships that could be construed as a potential conflict of interest.
